# COVID-19 Vaccination Efforts: Is Afghanistan Prepared?

**DOI:** 10.4269/ajtmh.21-0448

**Published:** 2021-08-31

**Authors:** Mohammad Faisal Wardak, Ali Rahimi, Attaullah Ahmadi, Shekiba Madadi, Shamim Arif, Aziz Mahmood Nikbin, Ghulam Ali Nazari, Ahmad Tariq Azizi, Sayed Hamid Mousavi, Don Eliseo Lucero-Prisno

**Affiliations:** ^1^ Medical Faculty, Herat University, Herat, Afghanistan;; ^2^ Medical Research Center, Kateb University, Kabul, Afghanistan;; ^3^ Medical Faculty, Ghalib University, Herat, Afghanistan;; ^4^ Afghanistan National Charity Organization for Special Diseases (ANCOSD), Kabul, Afghanistan;; ^5^ Department of Global Health and Development, London School of Hygiene and Tropical Medicine, London, United Kingdom

## Abstract

A country’s preparedness for a prompt and successful implementation of vaccination programs plays a pivotal role in disease control and prevention. As it stands now, Afghanistan seems to be ill-prepared to embrace a successful implementation of the COVID-19 vaccination program because of a spate of challenges. These include, but are not limited to, the insufficient number of vaccinators, a dearth of fully integrated functioning cold chain, challenging geographical barriers, cultural issues, insecurity, and protracted conflict. The COVID-19 infodemic along with vaccine mistrust in the country will lead to a pervasive public vaccine hesitancy in Afghanistan, which will present serious obstacles to the COVID-19 immunization efforts. The politicization of the Ministry of Public Health (MoPH) and the complaints of embezzlement and misuse of the pandemic aid have already eroded public trust during the pandemic. To ensure a large-scale and equitable distribution of COVID-19 vaccines, the cold chain infrastructure should be strengthened, and the immunization personnel trained. Antivaccination propaganda and misinformation should be tackled with effective communication approaches and effective community engagement, which consider culturally relevant messages appropriate to the culture and people. The allegations of corruption should be addressed to revive public trust in public health interventions, including COVID-19 vaccination.

COVID-19 is the most serious global health threat of our time, which continues to strain healthcare systems globally.^1^^,^^2^ Despite the international community’s concerted efforts, such as enforcing lockdowns, restrictions, and curfews and global collaboration on developing COVID-19 tests and vaccines, the pandemic has been spreading at a rapid pace with a worldwide cumulative count of 181,695,165 confirmed cases and 3,935,404 deaths by June 27, 2021.^3^ No approved specific treatment has been proposed for the management of the disease, thus requiring empirical approaches for now. The production and implementation of safe and scientifically certified vaccines are necessary to gain control of the disease through flattening the epidemic curve, that is, lowering COVID-19 hospitalizations, complications, and mortality. Twenty-one COVID-19 vaccines have been granted permission for emergency use by national regulatory authorities. At least one WHO-recognized stringent regulatory authority has approved at least six of them for emergency or full use (Oxford–AstraZeneca, Pfizer-BioNTech, Sinopharm-BBIBP, Moderna, Sinovac, and Janssen).^4^ UNICEF, in collaboration with the Pan American Health Organization (PAHO) Revolving Fund, procure COVID-19 vaccines as part of the COVID-19 Vaccines Global Access (COVAX) Facility, which is led by the Global Alliance for Vaccines and Immunizations (GAVI), the vaccine alliance, for 92 low- and lower-middle-income countries, including Afghanistan.^5^

Afghanistan has received COVID-19 vaccines under COVAX and through the financial support of the international community. The National Technical Team (Responsible Committee for COVID-19 Vaccination in Afghanistan) intends to distribute these vaccines first to people at high risk. Target groups in the order of priority are: 1) health workers, 2) teachers, 3) security personnel, 4) prisoners, 5) people with comorbidities, 6) people aged above 50, 7) nomadic people, 8) internally displaced people, 9) returnees from countries with high prevalence, 10) government employees who are working with crowds, and 11) people living in urban slums of big cities and for emergency uses.^6^^,^^7^

COVID-19 Vaccines Global Access aims to sponsor COVID-19 vaccine costs to 20% of the Afghanistan population in six phases by the end of 2022. For another 20% of the population in the next 2 years, the country will buy vaccines with the financial support of the World Bank and the Asian Development Bank. This means that by the end of 2022, nearly 40% of the population will be vaccinated.^6^ A plan to vaccinate the remaining 60% of the population is currently being developed.^6^^,^^7^ Afghanistan has received the first 500,000 doses of Oxford–AstraZeneca’s COVID-19 (Covishield) vaccine from India, a viral vector vaccine that consists of two doses with an interval of 4–12 weeks between doses. Therefore, the first stage of vaccination began on February 23, 2021. However, the first vaccines under the COVAX program arrived on March 8, 2021, consisting of 468,000 doses of Covishield vaccines.^8^ Additionally, other 700,000 doses of Sinopharm-BBIBP vaccines, inactivated virus double-doses COVID-19 vaccines, from China arrived in Afghanistan on June 12, 2021, after the vaccination process paused for a week because of vaccine shortage.^9^ A total of 582,128 (1.5%) of the Afghanistan population has received at least one dose of a COVID-19 vaccine, with 183,762 (0.5%) fully vaccinated as of June 22, 2021.^10^ In addition, 1.4 Million doses of Janssen COVID-19 vaccines, another viral vector but single-dose vaccines, helped by the United States also arrived in Kabul on June 9, 2021.^11^

Vaccine shortage caused Afghanistan’s national vaccination program to pause for a brief period beginning on June 6, 2021. This pause coincided with the spread of the third wave of COVID-19 in Afghanistan as a result of the arrival of the new Delta variant.^9^ The share of the Afghan government to services in the health sector is very low (around 5%). The sector hugely depends on foreign donors like the United States, European Union, and World Bank, which finance the health service delivery project in the country^7^^,^^12^ making foreign donations a decisive factor in strengthening the COVID-19 vaccination campaigns in Afghanistan.

Vaccination programs have been facing many challenges and barriers in Afghanistan for a long time now. Despite fundamental efforts, the country is one of the two remaining countries endemic to polio.^13^ The significance of a country’s preparedness for the successful implementation of vaccines to tackle a disease cannot be overemphasized. In this article, we aim to provide an analysis of Afghanistan’s preparedness for effective COVID-19 vaccination efforts and the challenges lying ahead. We also provide essential key recommendations on how to mitigate those challenges and barriers.

In Afghanistan, the primary factors for the failure of successful vaccination at the country level include inaccessible vaccination sites because of security concerns and geographical barriers, cultural constraints, vaccine illiteracy, and misconceptions.^13^^,^^14^ In addition, the dearth of sustainable cold chain, lack of infrastructure for power source, insufficient health facilities for immunization services, lack of effective service delivery, inadequate expert supervisors, low capacity of staff, and lack of skilled technicians are other issues that hamper national immunization efforts.^15^ These deficits in the immunization infrastructure result in distribution failures and lead to vaccine wastage, missed opportunities for vaccination, and inadequate immunization rates.^15^

Preparing an efficient cold chain system and service delivery to avoid vaccine wastage is a crucial issue. However, as of the present, there are no national cold chain maintenance plans, handbooks, manpower for vaccine delivery and for cold chain system, and skilled cold chain technicians.^7^^,^^15^ As such, the vaccine wastage rate is high in the country. For instance, the wastage of vaccines for Bacillus Calmette–Guérin (BCG) and Inactivated Polio Vaccine (IPV) is 86% and 28%, respectively.^15^ The transportation of vaccines also faces basic challenges such as lack of refrigerated vaccine vans, and having freelance drivers with insufficient information and skill of sensitive transportation of vaccines. There are also issues with cold chain space, electricity supply duration, spare parts, documentation of breakdown date and repair date, and shortage of cold chain equipment, which does not comply with GAVI data standards.^15^ As a result, Afghanistan does not get vaccines that require an ultracold chain (e.g., −70°C). The country has requested vaccines that fit its current cold chain system, which is +2 to +8°C. In addition, the country can store the vaccine up to −20°C at the national, regional, and provincial levels only.^6^^,^^7^ Viral vector vaccines such as Janssen and Covishield, and inactivated virus vaccines such as Sinopharm, which are now in use in Afghanistan, can be stored at 2–8°C.^6^^,^^8^^,^^9^

Afghanistan’s Ministry of Public Health (MoPH) should augment the cold chain supply for vaccines by developing a national cold chain maintenance plan and a national action plan for the immunization supply chain. It must specify transport standards to the transporter in the contracts and should have need-based refrigerated vaccine vans. Supervisors at vaccination sites should keep temperature monitoring records and review them regularly. Regular capacity building should be conducted for the staff and more technicians should be hired.^7^^,^^15^ Ministry of Public Health could also be supported by the private sector, which have the resources including their refrigerators and transportation vans. Certain measures can be taken by MoPH to prevent vaccine wastage, such as providing only the needed number of vaccines to rural areas with no refrigeration requirements and utilizing off-grid cooling equipment, that is, solar-powered refrigerators.

Low immunization coverage for other preventable diseases in Afghanistan implies possibly the same fate for COVID-19 immunization. Low immunization coverage is partially because of low access to vaccines in Afghanistan, mainly because of geographical constraints, that is, harsh weather and mountainous terrains. Provinces with cold climate such as Ghor, Badakhshan, Nooristan, and Kunar have a limited time frame by which vaccinations are possible to administer.^14^ Around 30% of the population have limited access to basic health services within a 2-hour travel radius hence, only 50% of children under 5 years of age have received the full suite of recommended vaccinations.^16^ As per government data, the health facility-based campaigns could only reach a maximum of only 20% of targeted children (sometimes even 3% in some high-risk areas of the southern region). This low coverage is mainly because of insufficient health facilities in some districts, difficult terrain to traverse, long distances, and lack of motivation among community members to travel long distances for vaccination. Vaccination coverage status is lower among children living in rural areas than in urban areas. The spread of the population and movement of Kochis (nomads) in provinces such as Badghis, Farah, Herat, and Ghor cause low immunization coverage as it is hard to track them because of their continuous wandering practices.^14^

Low vaccine coverage in harsh geographies can be solved by using animal transport to move supplies instead of vehicles and by deploying additional teams from local areas. In an event of a natural disaster like during wintertime (e.g., snowstorms and road blockades), MoPH should work on geographical prioritization and rescheduling the vaccination program.^7^ Special strategies for the provision of services should be developed for insecure and hard-to-reach areas such as expanding the mobile vaccine program staff. Another viable solution is the expansion of stationary facilities for immunization while considering the realistic situation on the ground for each province.^14^^,^^15^ There are 2,227 Expanded Program on Immunization (EPI) Centers in Afghanistan, which are all equipped with standard WHO prequalified cold chain equipment. To increase immunization coverage, the MoPH could also rely on its EPI centers, or its supplementary immunization activities (SIA), which require a slightly different implementation strategy than fixed EPI centers. It requires additional human resources especially in hard-to-reach areas to be recruited, trained, and deployed to carry out the interventions.^7^

Furthermore, the lack of trained human resources and skilled health workers to assist in the vaccination program poses difficulties for the proper implementation of vaccines. Most of the vaccinators are not well trained and refresher training is not provided systematically. In some cases, their salaries are not paid on time compelling them to leave their jobs. Low remuneration and incentives result in a high turnover of vaccinators. The national EPI manager estimates that only 10% of the vaccinators fulfill the required criteria—a 12-year basic education, complemented with a 3-month theoretical and 1-month practical training on vaccination. According to the data of health facilities, microplanning (training to identify priority communities, addressing barriers, and developing work plans with solutions) was found in only 14% of health facilities, and preservice training was provided to only 61% of EPI staff, particularly vaccinators.^15^

The National Technical Committee devised a combined strategy to tackle this problem using existing routine immunization vaccinators and the deployment of 2,000 additional new health workers (team of two persons: one male and one female). All vaccinators (existing and additional) will receive proper standard training. With the support of GAVI, the microplanning exercise is progressing and will be completed in 34 provinces by mid-2021.^7^ Competence-based management for recruitment should be implemented. As the World Bank and Asian Development Bank support Afghanistan’s COVID-19 vaccination process, MoPH may not face a financial crisis and could pay vaccinators fair amounts and on time. These approaches will relieve some of the pressure on the health system in Afghanistan.

Furthermore, infodemics about vaccines pose a serious threat to vaccination success.^17^ These misconceptions vary from the belief that vaccines cause sterility to the idea that vaccines are made of pork fats that are prohibited in Islam. A profusion of misinformation on numerous social media sites can easily lead to vaccine hesitancy and disease nonchalance. With the proliferation of the use of social media in Afghanistan, the value of immunization and vaccine safety has sparked an online debate, the spread of rumors, and misinformation.^18^ To combat the misinformation and infodemics, MoPH leadership should inform the public and correct the misunderstanding through media using risk communication techniques. In case of low demand and hesitancy, they should design and broadcast communication plans and specific messages to inform and encourage people.^7^ Antivaccination propaganda and misinformation should be tackled with effective communication approaches, mainly through social media, television networks, and trusted members of the society such as religious leaders and celebrities. This will improve vaccine literacy and the public perception of the vaccine.

Cultural constraints regarding women are another issue that inhibits full immunization coverage. In many places, men do not allow their women to be vaccinated by a male health worker. Females are confined to their homes having limited movement of freedom and cannot go to health centers unless they are accompanied by their *mahrams* (members of the family).^12^ Ministry of Public Health has a comprehensive plan to vaccinate all women and men in COVID-19 high-risk groups. Main actions to tackle gender disparity within the vaccination program include 1) raising awareness about the significance of both male and female immunization, and for community mobilizations, in the targeted groups through raising awareness and education of the general public; 2) provision of the number of vaccine doses needed; and 3) inclusion of women in the front line of health workers/vaccinators.^7^

Moreover, conflict-affected areas pose a big challenge to COVID-19 vaccination programs. The protracted conflict in Afghanistan, which is increasing the pressure on the healthcare system has been hindering the population from getting full immunization for a long time now. In May 2018, the antigovernment elements (such as the Taliban and Daesh) imposed a ban on house-to-house immunization; the ban was expanded in 2020, thus further restricting access to children who need to be vaccinated. The ban has had a major impact on polio immunization.^13^ Negotiation with antigovernment elements through community elders and influencers would be helpful to convince the locals to get vaccinated. MoPH could also consider recruiting and deploying more vaccinators familiar with the local circumstances from that area. However, in circumstances of significantly increased ongoing conflicts in specific locations—following the announcement of US and NATO withdrawal—rescheduling vaccination programs in conflict areas could be a viable approach.^7^

Corruption in Afghanistan is another rampant issue that has the potential to interfere with the immunization process. For example, corruption allegations against two former public health ministers have already increased distrust among the public and foreign donors. Increasing mistrust in the Afghan government because of the alleged COVID-19 fund mismanagement may also lead to significant vaccine denial. The real and perceived politicization of COVID-19 and related public health institutions, such as the MoPH, may set the stage for partisan vaccine allocation and distribution, fueling COVID-19 conspiracy theories.^19^ The Afghan government needs to follow and address the corruption allegations, and the healthcare community must avoid the political arena when conducting the vaccination campaign to revive public trust in public health interventions. Employment of apolitical entities for conducting vaccinations and forming public oversight committees at the province/district level reflecting the demographic makeup of the province/district can build public trust in the government and ensure fairness and equity in the distribution of vaccines.

Although the international assistance and cooperation play a crucial role in Afghanistan’s COVID-19 immunization coverage efforts, this process will not be without challenges. Low immunization coverage, weak vaccine logistics, insecurity, insufficiently skilled health workers, misinformation, hesitancy, and cultural issues will present obstacles against COVID-19 vaccination campaigns. Based on the current vaccine supply sources, Afghanistan is hugely dependent on donations and foreign aid. The solutions and recommendations provided in this article, if implemented properly, have the potential to render the whole vaccination campaign a success.

## References

[1] Dzushupov K Lucero-Prisno DE Vishnyakov D Lin X Ahmadi A, 2021. COVID-19 in Kyrgyzstan: navigating a way out. J Glob Health 11: 03020. 33643630 10.7189/jogh.11.03020 PMC7898654

[2] Lucero-Prisno DE Ahmadi A Essar MY Lin X Adebisi YA, 2020. Addressing COVID-19 in Afghanistan: what are the efforts and challenges? J Glob Health 10: 020341. 10.7189/jogh.10.020341 PMC7568006 33110541

[3] World Health Organization , 2021. *Status of COVID-19 Vaccines within WHO EUL/PQ Evaluation Process*. Available at: https://extranet.who.int/pqweb/sites/default/files/documents/Status_COVID_VAX_18May2021.pdf. Accessed June 27, 2021.

[4] COVID-19 Vaccine Tracker , 2021. *UNICEF to Lead Procurement and Supply of COVID-19 Vaccines in World’s Largest and Fastest Ever Operation of its Kind*. Available at: https://vac-lshtm.shinyapps.io/ncov_vaccine_landscape/. Accessed July 2, 2021.

[5] UNICEF , 2021. *UNICEF to Lead Procurement and Supply of COVID-19 Vaccines in World’s Largest and Fastest Ever Operation of its Kind.* Available at: https://www.unicef.org/afghanistan/press-releases/unicef-lead-procurement-and-supply-covid-19-vaccines-worlds-largest-and-fastest-ever. Accessed June 27, 2021.

[6] Afghanistan Government Media and Information Center , 2021. د عامې روغتیا وزارت خبري کنفرانس (ministry of public health press conference). Available at: https://youtu.be/jog40ge1sWk. Accessed June 27, 2021.

[7] Afghanistan Ministry of Public Health , 2021 *. National Plan for COVID-19 Vaccination in Afghanistan*. Available at: https://www.adb.org/sites/default/files/linked-documents/55012-001-sd-03.pdf. Accessed June 26, 2021.

[8] Afghanistan Ministry of Public Health , 2021. *The Second Cargo of AstraZeneca’s COVID-19 Vaccine Arrived in Kabul.* Available at: https://www.youtube.com/watch?v=eFsoYDWJduc. Accessed June 27, 2021.

[9] Afghanistan Ministry of Public Health , 2021. ۷۰۰ هزار دوز واکسین کرونا کمک کشور چین به افغانستان رسید (700,000 doses of Chinese corona vaccine arrived in Afghanistan). Available at: https://moph.gov.af/dr/%DB%B7%DB%B0%DB%B0-%D9%87%D8%B2%D8%A7%D8%B1-%D8%AF%D9%88%D8%B2-%D9%88%D8%A7%DA%A9%D8%B3%DB%8C%D9%86-%DA%A9%D8%B1%D9%88%D9%86%D8%A7-%DA%A9%D9%85%DA%A9-%DA%A9%D8%B4%D9%88%D8%B1-%DA%86%DB%8C%D9%86-%D8%A8%D9%87-%D8%A7%D9%81%D8%BA%D8%A7%D9%86%D8%B3%D8%AA%D8%A7%D9%86-%D8%B1%D8%B3%DB%8C%D8%AF. Accessed June 27, 2021.

[10] Our World in Data, Coronavirus (COVID-19) Vaccinations-Statistics and Research , 2021. *Our World in Data*. Available at: https://ourworldindata.org/covid-vaccinations. Accessed June 27, 2021.

[11] Afghanistan Ministry of Public Health , 2021. یک محمولە واکسین کرونا بە کمک کشور امریکا امروز بە کابل رسید (A shipment of US-assisted corona vaccine arrived in Kabul today). Available at: https://moph.gov.af/dr/%DB%8C%DA%A9-%D9%85%D8%AD%D9%85%D9%88%D9%84%D9%87-%D9%88%D8%A7%DA%A9%D8%B3%DB%8C%D9%86-%DA%A9%D8%B1%D9%88%D9%86%D8%A7-%D8%A8%D9%87-%DA%A9%D9%85%DA%A9-%DA%A9%D8%B4%D9%88%D8%B1-%D8%A7%D9%85%D8%B1%DB%8C%DA%A9%D8%A7-%D8%A7%D9%85%D8%B1%D9%88%D8%B2-%D8%A8%D9%87-%DA%A9%D8%A7%D8%A8%D9%84-%D8%B1%D8%B3%DB%8C%D8%AF. Accessed June 27, 2021.

[12] Qaderi S , 2021. Transfusion-dependent beta-thalassemia in Afghanistan: current evidence amid COVID-19 and future recommendations. Hematology 26: 432–434. 34130618 10.1080/16078454.2021.1938814

[13] Ahmadi A Essar MY Lin X Adebisi YA Lucero-Prisno DE, 2020. Polio in Afghanistan: the current situation amid COVID-19. Am J Trop Med Hyg 103: 1367–1369. 32861265 10.4269/ajtmh.20-1010 PMC7543832

[14] Mugali RR , 2017. Improving immunization in Afghanistan: results from a cross-sectional community-based survey to assess routine immunization coverage. BMC Public Health 17: 290. 28376806 10.1186/s12889-017-4193-z PMC5379688

[15] Regional WHO, Office for the Eastern Mediterranean , 2021. *EPI Review Mission in Afghanistan.* Published online 2017. Available at: http://www.emro.who.int/images/stories/afghanistan/afg_epi_review_report_final.pdf. Accessed June 27, 2021.

[16] United Nations Office for the Coordination of Humanitarian Affairs (OCHA) Afghanistan , 2021. *Humanitarian response plan Afghanistan 2018–2021*. Available at: https://www.who.int/health-cluster/countries/afghanistan/Afghanistan-Humanitarian-Response-Plan-COVID-19-June-2020.pdf. Accessed June 27, 2021.

[17] Farooq F Rathore FA, 2021. COVID-19 vaccination and the challenge of infodemic and disinformation. J Korean Med Sci 36: e78. 33724740 10.3346/jkms.2021.36.e78 PMC7961870

[18] UNICEF , 2021. *Polio eradication*. Available at. https://www.unicef.org/afghanistan/polio-eradication.

[19] Schoch-Spana M , 2020. The public’s role in COVID-19 vaccination: human-centered recommendations to enhance pandemic vaccine awareness, access, and acceptance in the United States. Vaccine. Published online. doi: 10.1016/j.vaccine.2020.10.059. 10.1016/j.vaccine.2020.10.059 PMC7598529 33160755

